# YY1 directly suppresses MYCT1 leading to laryngeal tumorigenesis and progress

**DOI:** 10.1002/cam4.1073

**Published:** 2017-05-09

**Authors:** Si‐Yao Qu, Yuan‐Yuan Sun, Yun‐Hui Li, Zhen‐Ming Xu, Wei‐Neng Fu

**Affiliations:** ^1^ Department of Medical GeneticsChina Medical UniversityShenyang110122China; ^2^ National Laboratory of Medical Molecular BiologyInstitute of Basic Medical SciencesChinese Academy of Medical Sciences & Peking Union Medical CollegeBeijing100005China; ^3^ Department of Laboratory MedicineNo. 202 Hospital of PLAShenyang110003China; ^4^ Department of OtolaryngologyNo. 463 Hospital of PLAShenyang110007China

**Keywords:** Apoptosis, laryngeal cancer, migration, MYCT1, proliferation, YY1

## Abstract

YY1 is a key transcription factor and plays different roles in various cancers. However, role and mechanism of YY1 in laryngeal cancer are still unknown. YY1 and MYCT1 mRNA and protein levels were detected by Real‐time RT‐PCR and Western Blot methods, respectively. Binding of YY1 to MYCT1 promoter was predicted and confirmed by bioinformatics and chromatin immunoprecipitation assays, respectively. MYCT1 promoter activity was assessed by dual luciferase assay system. Laryngeal cancer cell proliferation, migration, and apoptosis were evaluated by cell viability, colony formation, cell scratch assay, transwell assay, and flow cytometry methods, respectively. YY1 and MYCT1 were upregulated and downregulated at transcriptional level in laryngeal cancer, respectively, which showed a negative correlation between YY1 and MYCT1 expression in laryngeal cancer. Significantly higher expression of YY1 and lower expression of MYCT1 were found in laryngeal cancer tissues of patients with lymphatic metastasis than those without metastasis.YY1 directly bound to MYCT1 promoter region and inhibited its promoter activity. YY1 silence had similar biological functions as MYCT1 overexpression in repressiveness of proliferation and migration, and promotion of apoptosis in laryngeal cancer cells. However, the effects of YY1 silence were recovered by MYCT1 knockdown. YY1 promotes proliferation and migration with suppression of apoptosis via directly inhibiting MYCT1 in laryngeal cancer cells, suggesting that YY1 is a useful target as a potential oncogene in laryngeal cancer development and progression.

## Introduction

Human MYCT1 cDNA was first cloned using in silicon hybridization and molecular methods and previously named MTLC (c‐Myc target from laryngeal cancer cell) by our team 1. Studies have showed that MYCT1 plays an important role in tumorigenesis either as a tumor suppressor gene or an oncogene in different tumors, implying that MYCT1 function in cancer is tissue‐specific. For example, MYCT1 is downregulated and promotes apoptosis in laryngeal and gastric cancer 1, 2. However, MYCT1 is upregulated and enhances cancer cell viability in acute myeloid leukemia 3. Therefore, study on MYCT1 regulation network helps us to understand the tissue‐specific manner of MYCT1 in cancer.

At present, several transcription factors have been reported to be a direct regulator of MYCT1. In our and other studies, C‐MYC directly targets MYCT1 promoter region and regulates its expression 1, 4, 5, 6. We also found that MYCT1 promoter region including C‐MYC binding site is hypermethylated in laryngeal cancer, suggesting that the methylation status interferes with the binding of C‐MYC to MYCT1 leading to MYCT1 dysregulation in laryngeal cancer 7. Liddiard et al. discovered that the fusion protein RUNX‐ETO contributes to key aspects of the leukemic phenotypes via targeting MYCT1 3. In addition to C‐MYC and RUNX‐ETO, other transcription factors regulating MYCT1 expression are not reported.

YY1 (Yin‐Yang‐1) is a zinc‐finger transcription factor that belongs to the GLI‐Krüppel gene family 8 and abnormally expressed in lots of cancer such as colon cancer 9, non‐small‐cell lung cancer 10, and myeloma 11, indicating that it has key roles in carcinogenesis. However, any information of YY1 in laryngeal carcinogenesis is not reported at present. By prediction, we found that MYCT1 is a novel candidate of YY1 targets. Therefore, we speculate that YY1 functions in laryngeal cancer probably via MYCT1.

In this study, we assessed YY1 and MYCT1 expression in laryngeal cancer tissues. We also monitored binding ability of YY1 to MYCT1 and regulatory pattern between them. We then detected effects of YY1 knockdown on laryngeal cancer proliferation, migration, and apoptosis in the presence and absence of siMYCT1.

## Materials and Methods

### Cell culture and tissues

Hep‐2 cells (human laryngeal cancer) and HEK293T cells (human embryonic kidney) were bought from the Cell Biology Institute of Shanghai, Chinese Academy of Science. Hep2 and HEK293T cells were grown in complete RPMI 1640 medium (GIBCO, LA, CA) and Dulbecco's high glucose modified Eagle's medium (DMEM) (GIBCO), respectively, containing 10% fetal bovine serum (SERANA, SA, AG), 100 units/mL penicillin, and 100 *μ*g/mL streptomycin in a humidified atmosphere at 37°C with 5% CO2.

Thirty pairs of cancer and matched noncancer tissues from laryngeal cancer patients were obtained in the department of Otolaryngology, No. 463 Hospital of PLA. Lymphatic metastasis was found in seven patients. The tissues above were immediately frozen at −80°C after surgical operation. Pathological type of each tissue was confirmed by a pathologist. All patients gave their informed consent and the study was approved by the Research Ethics Committee of China Medical University (Shenyang, China).

### Real‐time RT‐PCR

Total RNA was extracted from cells and tissues using Trizol (Takara, Dalian, China) according to the manufacturer's instructions. Total cDNA was synthesized with Prime Script RT Master Mix Perfect Real Time kit (Takara). Quantitative PCR was performed using SYBR^®^ Premix Ex Taq^™^ II (Takara) according to the manufacturer's instructions. Primer sequences are as follows: MYCT1 Forward‐5′‐GCCAGAAAA CTTTTGGGAGGA‐3′; and Revere‐5′‐ATCCAGTTCTGTTGAGGCCG‐3′; YY1 Forward‐5′‐TTGCTCAGTCAACTAACCTGAAATC‐3′; and Revere‐5′‐GAGGCA TATTTATTCCCAATCACAC‐3′; *β*‐actin Forward‐5′‐TGGCACCCAGCACAATG AA‐3′; and Revere‐5′‐CTAAGTCATAGTCCGCCTAGAAGCA‐3′. PCR product was normalized to endogenous *β*‐actin and quantified using the 2^−ΔΔCt^ method in relation to the control.

### Chromatin immunoprecipitation (ChIP)

Hep2 cells were crosslinked with 1% formaldehyde at 37°C for 10 min when cell density was estimated to reach about 80% confluence. ChIP assay was performed as described on the reference of EZ‐CHIP Reagent kit (Millipore, Billerica, MA). DNA‐protein complex of chromatin fragments was precipitated by anti‐YY1 antibody (Abcam, Cambridge) or anti‐IgG antibody (Santa Cruz, CA). DNA was then eluted and extracted with phenolchloroform and subjected to PCR. MYCT1 promoter‐specific primers were used to amplify the YY1 binding regions. Sequences for YY1 primers are as follows: Forward‐5′‐GAGGTCAGGCCTAGTTC ATG‐3′, and reverse‐5′‐CTTAGTCTCGCTCTGTCGC‐3′. PCR products were separated by electrophoresis on 2% agarose gel, stained with ethidium bromide and photographed.

### Gene transfection

pGFP‐MYCT1 vector, pGFP‐empty vector, and small RNAs including siMYCT1 RNA, siYY1 RNA, and negative control RNA were obtained from GENECHEM (Shanghai, China). The small RNA sequences are as follows: siYY1‐5′‐GACGACU ACAUUGAACAATT‐3′; siMYCT1‐5′‐GCUGUGAACGUCGAAGCAATT‐3′; negative control RNA‐5′‐UUCU CCGAACGUGUCACGUTT‐3′.

Cells cultured in six‐well plates were transfected with 2 *μ*g of vector or 100 nM of small RNA into Hep2 cells using jetPRIME^®^ in vitro DNA & siRNA transfection kit (Polyplus Transfection,Illkirch, France) following the manufacturer's instructions. Medium was replaced at 4 h and cells harvested at 48 h posttransfection were used for the following experiments. Each experiment was performed in triplicate.

### Luciferase assay

Luciferase reporter constructs containing wild‐type MYCT1 promoter (−648/−400, MYCT1‐W) and mutant‐type MYCT1‐Mu were obtained from GENECHEM. MYCT1‐Mu containing the presumed YY1 binding site (−571 to −598) was mutated from CAACATGGTGGAACTCCATCTCTAGTA to ACCACGTTGTTCCAGAACG AGAGCTGC.

Cells cultured in 24‐well plates were transfected with 1 *μ*g of MYCT1‐W or MYCT1‐Mu luciferase reporter and 20 ng of the Renilla luciferase reporter vector (GENECHE). After transfection for 48 h, cells were harvested in 100 mL of Passive Lysis Buffer (Promega, Madison, WI) and luciferase assay was performed using the Dual Luciferase Assay System (Promega) according to the manufacturer's instructions. Relative luciferase activity was calculated as the ratio of luciferase activity to Renilla luciferase activity. Each experiment was performed in triplicate.

### Western blotting

Protein was extracted from cells using RIPA cell lysis buffer (Beyotime, Shanghai, China) according to the manufacturer's instructions. Protein concentration was measured using the BCA protein assay (Beyotime). Immunoblotting was conducted using anti‐MYCT1 (1:300 dilution; Abcam) and anti‐*β*‐actin (1:1000 dilution; Proteintech, Wuhan, China) primary antibodies, and horseradish peroxidase conjugated against mouse or rabbit IgG (1:2000 dilution; ZhongShan, Guangdong, China) secondary antibody, respectively. Signals were detected with ECL Plus (Beyotime) according to the manufacturer's instructions.

### Cell viability assay

Cells were grown in six‐well plates to about 70% confluence and transfected with siYY1, siMYCT1, MYCT1, siYY1 + siMYCT1, control small RNA or empty vectors. 4‐5 × 10^3^ of Hep2 cells were seeded into 96‐well plates at 4 hr posttransfection and cultured for 1, 2, 3, and 4 d, respectively. Absorbance at 450 nm was then measured after incubation of the cells with 10 *μ*L of CCK8 dye (Kaygene) at 37°C for 2 h. Cell growth curve was constructed using OD450 nm as ordinate axis.

### Colony formation assay

Cells were grown in six‐well plates to about 70% confluence and transfected with siYY1, siMYCT1, MYCT1, siYY1 + siMYCT1, control small RNA or empty vectors. 4–5 × 10^3^ of Hep2 cells were seeded into six‐well plates at 4 hr posttransfection and allowed to grow until visible colonies formed. After 4 ~ 6 d, colonies were fixed with methanol, stained with hematoxylin and photographed under a microscope.

### Apoptosis assay

Cells were grown in six‐well plates to about 70% confluence and transfected with siYY1, siMYCT1, MYCT1, siYY1 + siMYCT1, control small RNA or empty vectors. Cells were digested and collected at 48 hr posttransfection. Cells were then washed with PBS twice, treated by Annexin V‐PE/7‐AAD Apoptosis Detection Kit (KeyGEN, Nanjing, China) according to the manufacturer's instructions, and analyzed with a Flow Cytometer (FACS calibur, Becton Dickinson, Franklin Lakes, NJ).

### Cell scratch assay

Cells were cultured in six‐well plates to about 70% confluence and transfected with siYY1, siMYCT1, MYCT1, siYY1 + siMYCT1, control small RNA or empty vector. A scratch was made in the monolayer using a P1000 pipette tip (Greystone Biosciences, USA). Images were recorded and analyzed at 0 h and 48 h.

### Transwell assay

Hep2 cells were cultured in six‐well plates to about 70% confluence and transfected with siYY1, siMYCT1, MYCT1, siYY1 + siMYCT1, control small RNA or empty vector. Cells were then detached and resuspended in serum‐free medium, and seeded to the upper chambers; 500 *μ*L of medium containing 10% FBS was added to the lower chambers.

After incubation at 37°C for 24 h, cells remaining attached to the upper surface of the filters were carefully removed with cotton swabs. Filters were then fixed with methanol and stained with hematoxylin and eosin. Cells that invaded to the underside of the filter were counted.

### Statistical analysis

Each experiment was performed at least three times. All data were expressed as mean ± standard error of the mean (SEM) and analyzed by SPSS program (IBM SPSS Statistics version 16.0). YY1 and MYCT1 expression levels were analyzed by paired sample *t*‐test. Correlation analysis between of YY1 and MYCT1 expression was performed by bivariate correlation. Statistical significance was indicated as * (*P* < 0.05).

## Results

### YY1 is negatively correlated with MYCT1 at transcription level in laryngeal cancer

**Figure 1:**
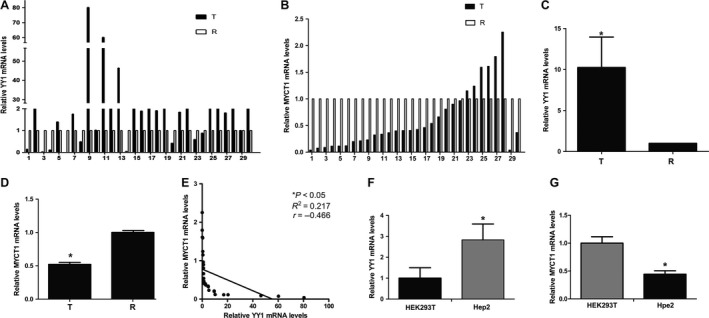
Association of YY1 and MYCT1 mRNA levels in laryngeal cancer. (A) General mRNA level of YY1 in laryngeal cancer tissues. (B) General mRNA level of MYCT1 in laryngeal cancer tissues. (C) Statistic analysis of YY1 mRNA level in laryngeal cancer tissues. (D) Statistic analysis of MYCT1 mRNA level in laryngeal cancer tissues. (E) Correlation between YY1 and MYCT1 mRNA levels in laryngeal cancer tissues. (F). Statistic analysis of YY1 mRNA level in laryngeal cancer cells. (G). Statistic analysis of MYCT1 mRNA level in laryngeal cancer cells. T and R indicate cancer tissues and paired noncancer tissues, respectively. Symbol “*” indicates *P* < 0.05.

Real‐time RT‐PCR results indicated that high YY1 and low MYCT1 mRNA levels in laryngeal cancer tissues were observed in 19 of 30 (63.33%) and 24 of 30 (80%) cases (Fig. 1A and B), respectively. Statistically, YY1 and MYCT1 were significantly upregulated and downregulated at transcriptional level in laryngeal cancer tissues compared to matched normal tissues, respectively (*P* < 0.05, Fig. 1C and D). Statistic analysis result displayed that there was a negative correlation between YY1 and MYCT1 mRNA levels (*P* < 0.05, Fig. 1E). Real‐time RT‐PCR results also showed that YY1 and MYCT1 mRNA levels were significantly higher and lower in Hep2 cells than those in HEK293T cells (*P* < 0.05, Fig. 1F and G), respectively, further suggesting the negative correlation between YY1 and MYCT1 at transcription level in laryngeal cancer.

**Figure 2:**
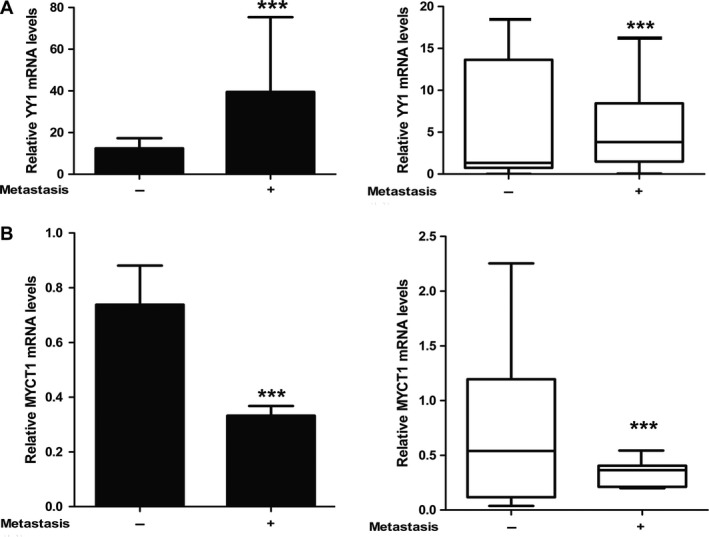
Association of YY1 and MYCT1 mRNA levels in laryngeal cancer tissues of the patients with lymphatic metastasis. (A) Statistic analysis of YY1 mRNA level in laryngeal cancer tissues from patients with or without lymphatic metastasis, respectively. (B) Statistic analysis of MYCT1 mRNA level in laryngeal cancer tissues from patients with or without lymphatic metastasis, respectively. Symbols “+”and “‐” display laryngeal cancer tissues from patients with or without lymphatic metastasis, respectively. Symbol “***” indicates *P* < 0.001.

We also analyzed the relationships between YY1 or MYCT1 mRNA levels and metastasis in the cancer tissues from laryngeal cancer patients with or without lymphatic metastasis. As results, we found that cancer tissues displayed a significantly higher YY1 level in patients with metastasis than those without metastasis (*P* < 0.01, Fig. 2A). However, there was a significantly lower MYCT1 level in the cancer tissues from patients with metastasis than those without metastasis (*P* < 0.01, Fig. 2B). These results imply that both YY1 upregulation and MYCT1 downregulation are related to laryngeal cancer metastasis.

### YY1 directly suppresses MYCT1 transcription activity in vitro

**Figure 3:**
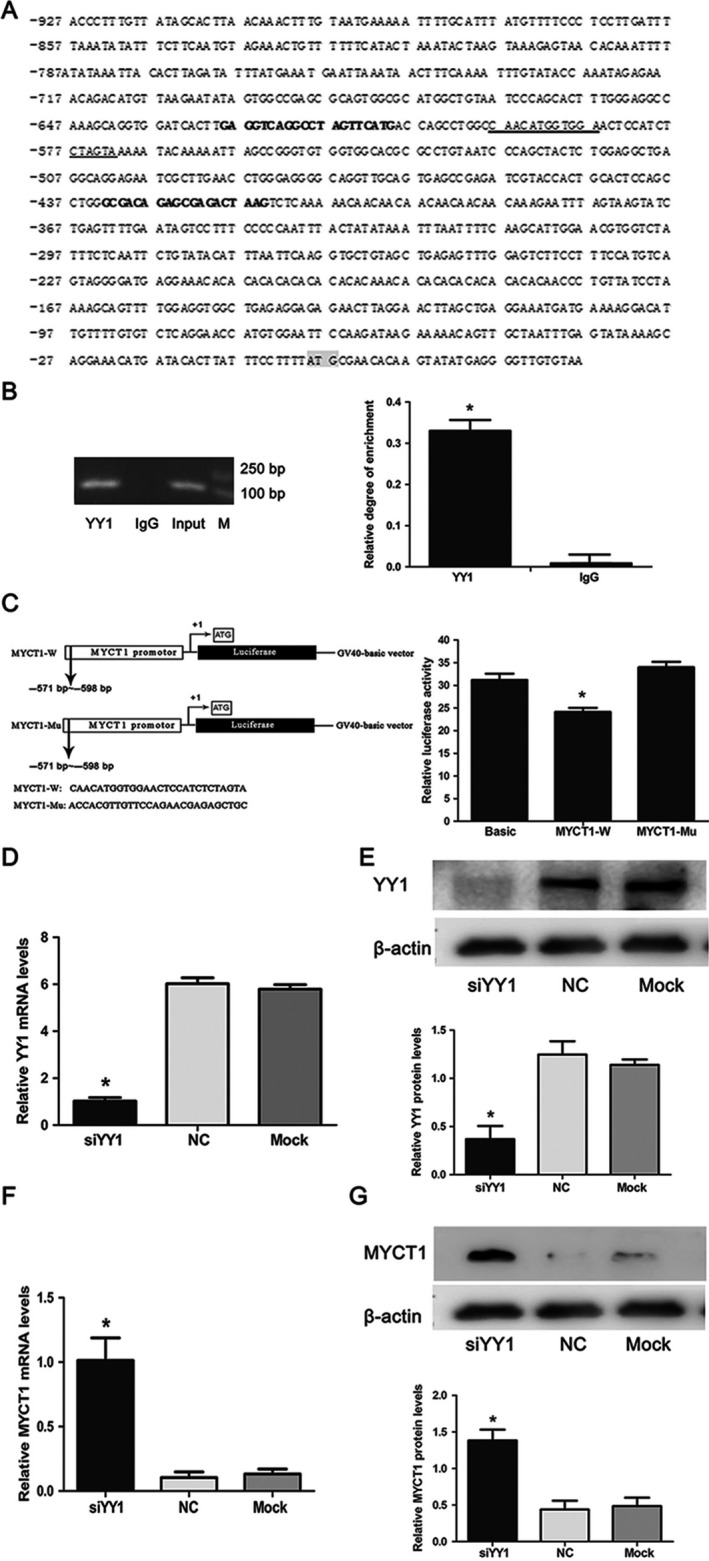
Regulatory manner of YY1 on MYCT1 promoter. (A) Prediction of YY1 binding site in MYCT1 promoter region. YY1 binding sequence within MYCT1 promoter was underlined. Bold letters indicate the PCR primer sequences for amplifying YY1 binding site in ChIP assay. (B) Binding detection of YY1 to MYCT1 promoter region by ChIP assay. PCR product containing YY1 binding site is 215 bp in length. (C) Luciferase activity assay of the MYCT1 gene promoter spanning YY1 binding site. Left, schematic diagram of the luciferase reporter constructs of MYCT1 promoter containing wild (MYCT1‐W) and mutant (MYCT1‐MU) YY1 binding site, respectively. Right, luciferase activity of MYCT1 promoter. (D). YY1 mRNA level in Hep2 cells transfected by siYY1. (E). YY1 protein level in Hep2 cells transfected by siYY1. (F). MYCT1 mRNA level in Hep2 cells transfected by siMYCT1. (G). MYCT1 protein level in Hep2 cells transfected by siMYCT1. NC and Mock are negative and black controls, respectively. Symbol * indicates *P* < 0.05.

As predicted by bioinformatics, there was a YY1 binding site in MYCT1 promoter, suggesting that MYCT1 is a potential target of YY1 (Fig. 3A).

ChIP assay result displayed that the binding sequence of YY1 in MYCT1 promoter was amplified based on chromatin fragment precipitated by anti‐YY1 antibody, implying that YY1 directly binds MYCT1 promoter in vitro (Fig. 3B). Luciferase reporter assay result revealed that luciferase activity of wild‐type MYCT1 promoter including YY1 binding site was significantly lower than that of mutant‐type in Hep2 cells (Fig. 3C). As shown in Figure 3D and E, siYY1 significantly repressed YY1 expression both at mRNA and protein levels in Hep2 cells, respectively, suggesting that YY1 is successfully knocked down. Meanwhile, we found that MYCT1 mRNA and protein expression levels were significantly increased after YY1 knockdown in Hep2 cells compared to the controls (*P* < 0.05, Fig. 3F and G).

Taken together with the negative correlation between YY1 and MYCT1 mRNA levels, these results indicate that YY1 directly and negatively regulates MYCT1 transcription activity in laryngeal cancer.

### Functions of YY1 knockdown in laryngeal cancer cells are reversed by MYCT1 knockdown

**Figure 4:**
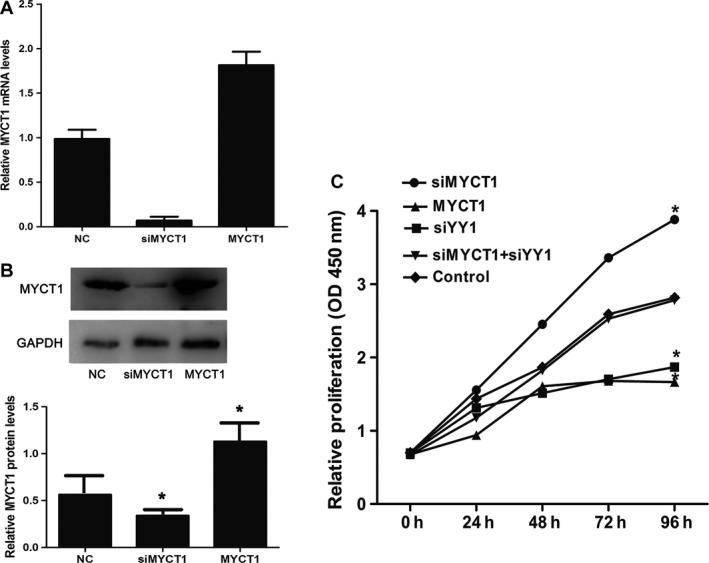
Effect of YY1 knockdown on laryngeal cancer cell viability via targeting MYCT1. (A) MYCT1 mRNA level in Hep2 cells transfected by MYCT1 and siMYCT1, respectively. (B) MYCT1 protein level in Hep2 cells transfected by MYCT1 and siMYCT1. (C). Effect of YY1 knockdown on laryngeal cancer cell viability. NC stands for negative control. Symbol * indicates *P* < 0.05.

As shown in Figure 4A and B, siMYCT1 and MYCT1 overexpression significantly reduced and boosted the MYCT1 gene expression both at mRNA and protein levels, respectively, suggesting that transfection is successful.

**Figure 5:**
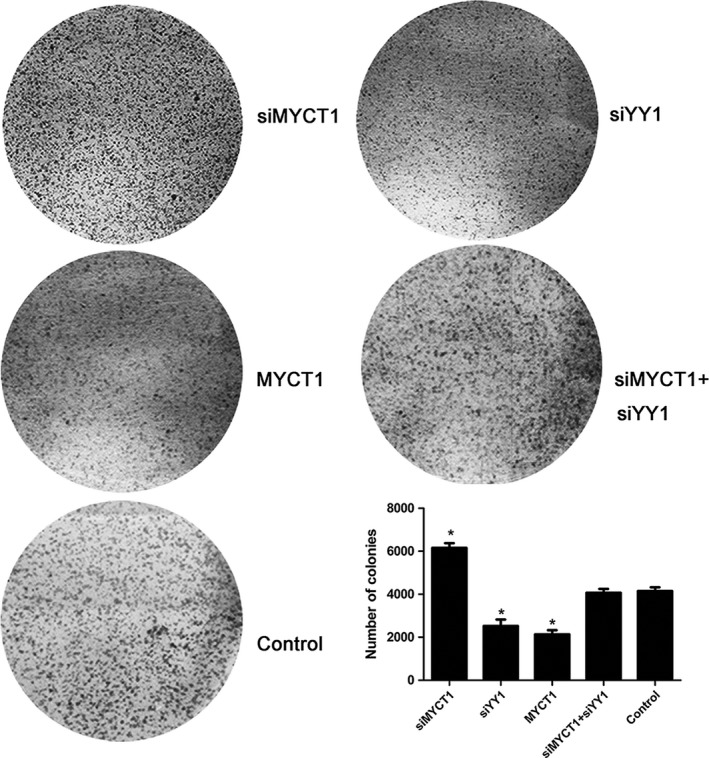
Effect of YY1 knockdown on laryngeal cancer colony formation via targeting MYCT1. NC indicates negative control. Symbol * indicates *P* < 0.05.

Similar to MYCT1 overexpression and contrary to MYCT 1 silence, YY1 knockdown significantly decreased laryngeal cancer cell proliferation (*P* < 0.05, Fig. 4C) and cloning formation compared to the control (*P* < 0.05, Fig. 5).

**Figure 6:**
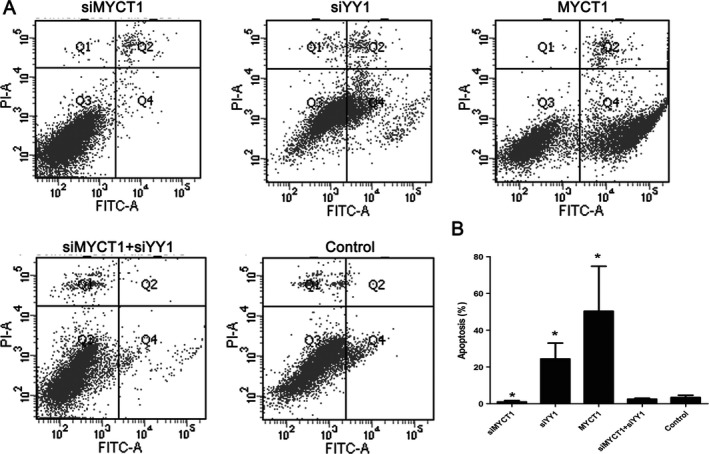
Effect of YY1 knockdown on laryngeal cancer apoptosis via targeting MYCT1. NC represents negative control. Symbol * indicates *P* < 0.05.

Contrary to MYCT1 knockdown, YY1 knockdown and MYCT1 overexpression significantly increased laryngeal cancer cell apoptosis compared to the control (*P* < 0.05, Fig. 6).

**Figure 7:**
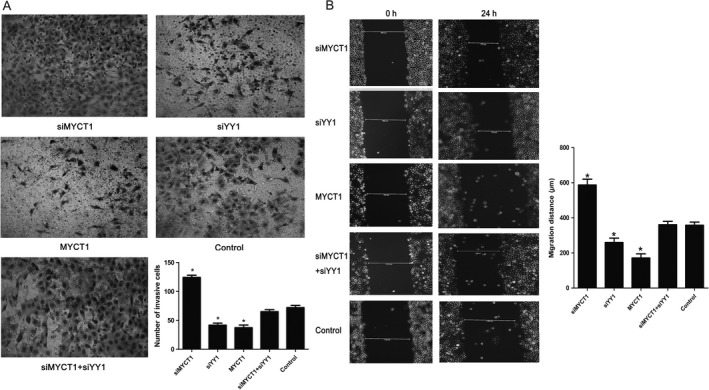
Effect of YY1 knockdown on laryngeal cancer cell migration via targeting MYCT1. (A) Effect of YY1 knockdown on laryngeal cancer cell migration by Transwell experiment. (B) Effect of YY1 knockdown on laryngeal cancer cell migration by scratch assay. NC displays negative control. Symbol * indicates *P* < 0.05.

Scratch and Transwell assay results displayed that YY1 knockdown and MYCT1 overexpression significantly reduced Hep2 cell migration ability compared to the controls (*P* < 0.05, Fig. 7A and B).

In order to confirm whether YY1 functions via MYCT1, we performed recovery experiment in Hep2 cells when cotransfected with siYY1 and siMYCT1. As revealed in Figures 4C–7, the effects of YY1 knockdown on laryngeal cancer proliferation, apoptosis, and migration were significantly reversed by MYCT1 knockdown compared to the controls (*P* < 0.05), respectively, implying that YY1 regulates laryngeal cancer proliferation, apoptosis, and migration by directly repressing MYCT1.

## Discussion

It is well‐known that oncogene overexpression and tumor suppressor underexpression are common events in cancer. Normal cells developing progressively to a neoplastic state undergo multiple processes in which 10 hallmarks of cancer, such as proliferation, apoptosis, and migration are involved 12, 13.

As a key transcription factor, YY1 regulates its target gene transcription in a very complex manner. YY1 can activate transcription of oncogenes and tumor suppressors, such as c‐Myc 14, c‐Fos 15, ERBB2 16, cyclooxygenase‐2 17, OTX2 18, Msx2 19, and Bax 20. YY1 can also repress transcription of oncogenes and tumor suppressors including Has2 21, HIF‐2*α* 22, PVT1 23, CEBPD 24, p16(INK4a) 25, and RB 26 etc.

In the study, we confirmed that MYCT1 is a novel target of YY1 and is negatively regulated by YY1 in laryngeal cancer. We also found that YY1 and MYCT1 are upregulated and downregulated in laryngeal cancer, respectively, implying that both genes participate in laryngeal carcinogenesis. Similar to our results, YY1 is overexpressed in most cancers, such as breast cancer 16, prostate carcinoma 27, ovarian cancer 28, brain cancer 29, acute myeloid leukemia 30, osteosarcoma 31, gastrointestinal cancer 32, cervical cancer 33, and hepatoblastoma 34.

Based on the different targets, YY1 has different roles in carcinogenesis. For example, YY1 promotes apoptosis via activating Bax and represses invasion and metastasis by downregulating MMP10 in pancreatic cancer cells, suggesting that it is a tumor suppressor 20, 35. However, YY1 plays an oncogenic role in some cancers such as melanoma. Study has shown that YY1 knockdown inhibits melanoma cell proliferation, cell cycle progression, migration, and invasion through a direct target of miR‐9 36. Similar to MYCT1, YY1 may act as an oncogene or a tumor suppressor in different cancer types.

In our study, YY1 silence and MYCT1 overexpression suppress proliferation and migration and promotes apoptosis in Hep2 cells, while siMYCT1 could rescue the effects of YY1 knockdown. We also found that high level of YY1 and low level of MYCT1 in laryngeal cancer tissues are correlated with metastasis, suggesting that both YY1 and MYCT1 genes might take part in laryngeal cancer progression. Taken together, we speculate that YY1 plays a potentially oncogenic role via directly inhibiting MYCT1 expression in laryngeal cancer genesis and progression.

Next, how MYCT1 functions in laryngeal cancer development is a key issue to be solved. In our previous study, we obtained series of differentially expressed genes using MYCT1 cDNA array assay (data not shown), which will help us to further study the molecular mechanism of MYCT1 in regulation of laryngeal cancer cell proliferation, migration, and apoptosis in the future. In addition, why YY1 is upregulated in laryngeal cancer is also required for investigation in our future study.

## Conclusions

YY1 promotes proliferation, migration with suppression of apoptosis via directly inhibiting MYCT1 in laryngeal cancer, suggesting that YY1 is a useful target as a potential oncogene in laryngeal cancer development and progression.

## Conflict of Interests

The authors declare that they have no competing interest.
